# Bispecific c-Met/PD-L1 CAR-T Cells Have Enhanced Therapeutic Effects on Hepatocellular Carcinoma

**DOI:** 10.3389/fonc.2021.546586

**Published:** 2021-03-10

**Authors:** Wei Jiang, Tao Li, Jiaojiao Guo, Jingjing Wang, Lizhou Jia, Xiao shi, Tingting Yang, Ruonan Jiao, Xin Wei, Zhenqing Feng, Qi Tang, Guozhong Ji

**Affiliations:** ^1^ Department of Gastroenterology, The Second Affiliated Hospital of Nanjing Medical University, Nanjing, China; ^2^ Jiangsu Key Lab of Cancer Biomarkers, Prevention and Treatment, Collaborative Innovation Center for Cancer Personalized Medicine, Nanjing Medical University, Nanjing, China; ^3^ Key Laboratory of Antibody Technique of National Health Commission, Nanjing Medical University, Nanjing, China; ^4^ Department of Pathology, Nanjing Medical University, Nanjing, China

**Keywords:** hepatocellular carcinoma, c-Met, PD-L1, CAR-T cell, antigen relapse effects

## Abstract

T cells expressing chimeric antigen receptors, especially CD19 CAR-T cells have exhibited effective antitumor activities in B cell malignancies, but due to several factors such as antigen escape effects and tumor microenvironment, their curative potential in hepatocellular carcinoma has not been encouraging. To reduce the antigen escape risk of hepatocellular carcinoma, this study was to design and construct a bispecific CAR targeting c-Met and PD-L1. c-Met/PD-L1 CAR-T cells were obtained by lentiviral transfection, and the transfection efficiency was monitored by flow cytometry analysis. LDH release assays were used to elucidate the efficacy of c-Met/PD-L1 CAR-T cells on hepatocellular carcinoma cells. In addition, xenograft models bearing human hepatocellular carcinoma were constructed to detect the antitumor effect of c-Met/PD-L1 CAR-T cells *in vivo*. The results shown that this bispecific CAR was manufactured successfully, T cells modified with this bispecific CAR demonstrated improved antitumor activities against c-Met and PD-L1 positive hepatocellular carcinoma cells when compared with those of monovalent c-Met CAR-T cells or PD-L1 CAR-T cells but shown no distinct cytotoxicity on hepatocytes *in vitro*. *In vivo* experiments shown that c-Met/PD-L1 CAR-T cells significantly inhibited tumor growth and improve survival persistence compared with other groups. These results suggested that the design of single-chain, bi-specific c-Met/PD-L1 CAR-T is more effective than that of monovalent c-Met CAR-T for the treatment of hepatocellular carcinoma., and this bi-specific c-Met/PD-L1 CAR is rational and implementable with current T-cell engineering technology.

## Introduction

Hepatocellular carcinoma (HCC) is the most common primary liver malignancy (1). It is the third most common cancer and the second most common cause of death in China (2). Currently, surgical resection is considered the first choice for patients. However, most HCC patients are in advanced stages at the time of initial diagnosis. Even after surgery, the tumor recurrence rate is about 55% within two years (3). Other traditional therapies such as chemotherapy, microwave ablation and radiofrequency therapy also have little effects, with a 5-year survival rate of only 10% (4). Sorafenib, the most representative molecular targeted drug for HCC, can easily cause a widespread range of adverse reactions due to its multi-target effects, and can only prolong the overall survival by 2 to 3 months (5, 6). Therefore, more effective therapeutic strategies are required to prolong the survival of HCC patients and improve their quality of life.

Currently, CAR-modified adoptive T-cell therapies have achieved encouraging clinical results in the treatment of hematological malignancies (7, 8). For solid tumors, CAR-T treatment has also achieved progress, including glioblastoma (9), mesothelioma (10), osteosarcoma (11) and colorectal cancer (12). Although Gao et al. reported that targeting Glypican 3 (GPC3) showed good response in GPC3-expressing HCC, research regarding HCC is still limited (13). Nowadays, antigen escape is a significant factor that affects the efficacy of CAR-T cells therapy (14). The solution to this problem is to construct a bi-specific CAR that could target two antigens, this new CAR structure is also called tandem-CAR (15, 16). This bi-specific CAR has made great a breakthrough and significantly improved the efficacy of CAR-T cells therapy (17).

c-Met, also known as hepatocyte growth factor receptor (HGFR) or tyrosine protein kinase Met. The expression of c-Met has been abnormally increased in HCC patients (18, 19), and suppression of c-Met can inhibit cell proliferation, invasion and epithelial-mesenchymal transition and other biological processes of tumor (20, 21). In addition, c-Met overexpression usually leads to poor patient prognosis (22, 23). Current therapies targeting c-Met have been reported in several tumors (24–26). A series of studies have suggested that c-Met inhibitors can inhibit the proliferation and invasion of c-Met-positive HCC (27, 28). Previous studies have demonstrated that c-Met CAR had superior inhibitory effects on the growth of mesothelioma and breast cancer *in vitro* and *in vivo* (10, 29). Previously, we have reported successful construction of high-affinity antibodies against human c-Met (30). In addition, our previous studies have shown that anti-human c-Met IgG-conjugated antibody drugs exhibit superior effects on c-Met positive HCC *in vitro* and *in vivo* (31, 32). Our previous study constructed c-Met monovalent CAR successfully, then transfected it into T cells for subsequent experiments (33). Therefore, c-Met may be a promising target for HCC immunotherapy.

PD-L1 (also known as B7-H1 or CD274) is a cognate ligand for programmed cell death protein 1 (PD-1) which is overexpressed in a variety of tumors (34–36). The binding of PD-1 and PD-L1 can inhibit T cell activation, proliferation, and survival (37). PD-L1 monoclonal antibody has been approved for the treatment of melanoma (38), bladder cancer (39) and lymphoma (40). PD-L1 expression was found to be significantly negatively correlated with the prognosis of patients with hepatocellular carcinoma (41, 42). In addition, blocking the binding of PD-1 to PD-L1 restores the function of CD8 + TIL cells (43). These data suggest that PD-L1 is likely to be a new target for the treatment of HCC.

In this study, the bispecific c-Met/PD-L1(CP) CAR-T cells were constructed successfully. In addition, the results shown that the bispecific CP CAR-T cells would offset antigen escape, and had more effective anti-tumor effects on HCC cells *in vitro* and *in vivo* than c-Met monovalent CAR-T cells.

## Materials and Methods

### Cell Lines

Human HCC cell lines HepG2 and MHCC-97, human liver normal epithelial cells LO2 were purchased from Shanghai Institute of Biochemistry and Cell Biology (SIBCB), Chinese Academy of Sciences (Shanghai, China). Human embryonic kidney (HEK)-293T cell line was provided by the Key Laboratory of Antibody Technology of National Health Committee of Nanjing Medical University. The HepG2 cell line expressing firefly luciferase (HepG2-fLuc) and shRNA inhibiting c-Met (HepG2^lo^) HepG2 cells were established by lentiviral transduction. All the cell lines mentioned above were in cultured Dulbecco’s Modified Eagle Medium (DMEM) (Gibco, USA) containing 10% fetal calf serum (FBS) (ScienCell, USA), 100 μg/ml penicillin and 100 μg/ml streptomycin (Gibco, USA). All cell lines were cultured at 37°C in a 5% CO_2_ incubator.

### Recombination of c-Met/PD-L1 (CP) CAR

**Figure 1:**
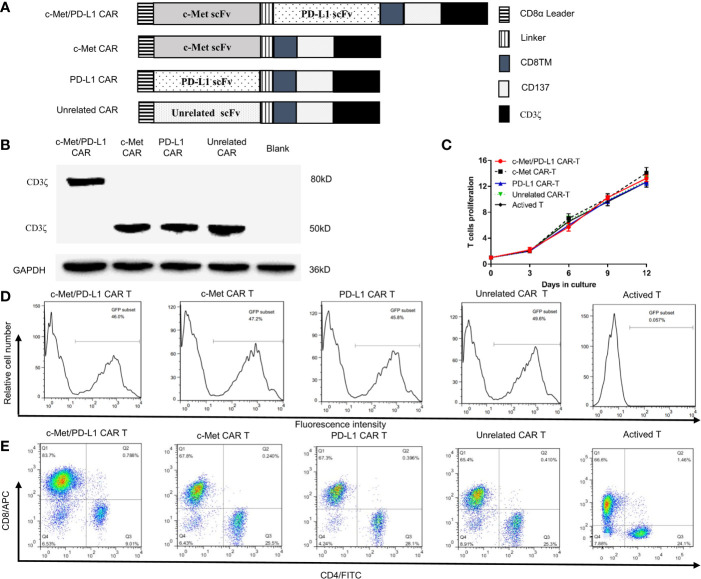
Construction and identification of CP CAR-T cells. **(A)** Schematic illustration of a lentiviral vector encoding CARs. **(B)** Expression of CD3ζ in 293T cells transfected with different CARs was analyzed by Western blot analysis. **(C)** *In vitro* proliferation rate of CAR-T cells. **(D)** Transduction efficiency of chimeric receptor expression on T cells was detected using flow cytometry. **(E)** The phenotype of CAR-expressing T cells was detected by flow cytometry after staining with anti-human CD4-FITC and anti-human CD8-APC. Data are shown as mean ± SD.

The anti-c-Met scFv and anti-PD-L1 scFv was derived from Fab (31), which has the ability to bind to c-Met or the PD-L1 extramembrane domain. CP CAR is designed to contain the human CD8α leader sequence, human anti-human c-Met scFv (VL-VH), human anti-human PD-L1 scFv (VH-VL), CD8α transmembrane domain (CD8TM), and the cytoplasmic domains of CD137 and CD3ζ (**Figure 1A**). The variable regions of anti-c-Met Fab (c-Met VH and c-Met VK) and anti-PD-L1 Fab (PD-L1 VH and PD-L1 VK) were obtained by PCR, and the amino sequences for this scFv were provided in **Supplementary Table 1**. The nucleic acid sequences of the c-Met VH, c-Met VK, PD-L1 VH, PD-L1 VK, and the intracellular signaling domain of CAR were spliced ​​by overlap PCR. The lentiviral expression plasmid pCDH-CMV-MCS-EF1α-CopGFP was cleaved by the restriction enzymes Not I and Xba I, and the above fragments were cloned into a linearized lentiviral expression vector using infusion PCR. The correctness of the recombinant expression vector was confirmed by enzyme cleavage and sequencing analysis. The c-Met CAR, PD-L1 CAR and unrelated CAR were structured in a similar way. Our earlier study had proved that bispecific c-Met/PD-L1 CAR can specifically recognize and bind to c-Met and PD-L1 at the protein and cell levels (44).

### CP CAR Lentivirus Production and Transduction

For the production of CP CAR lentivirus, CP CAR vector, plasmid psPAX2, and plasmid pMD2G were co-transfected into 293T cells using polyethylenimine transfection reagent (Invitrogen, USA). The supernatant containing the lentivirus was collected and concentrated 100 times by ultracentrifugation (Amicon Ultra 100 kD, USA). 293T transfected with c-Met CAR, PD-L1 CAR, or Unrelated CAR, and untransfected 293T were used as controls, and the expression of CD3ζ was detected by WB assays to detect the expression of the transfected CAR plasmid. The titer of the lentivirus was counted by the LaSRT method and observed by fluorescence microscopy (**Supplementary Figure 1A**).

### Preparation of CP CAR-T Cells

Human peripheral blood mononuclear cells (PBMCs) isolated from human donors were provided by the second affiliated hospital of Nanjing Medical University, and this study was approved by the ethical review board. First, peripheral blood mononuclear cells (PBMCs) were *via* discontinuous density gradient centrifugation based on diluted Ficoll-Paque PLUS (GE Healthcare Life Sciences, USA) and activated with a final concentration of 1 μg/ml anti-CD3/CD28 monoclonal antibody for subsequent use. For lentivirus transduction, non-tissue culture treated 24-well plates were precoated with 10 μg RetroNectin (Takara, Japan) in 500 µl PBS for 2 h at room temperature. The wells were then washed with HBSS (Gibco, USA) and incubated with CP lentivirus for 2 h at 32°C. Then, 1 x 10^6^ anti-CD3/CD28 activated T cells were cultured in the coated wells. After 48 h, the transduced T cells were transferred to new plates. Similarly, c-Met CAR-T, PD-L1 CAR-T, Unrelated CAR-T were obtained. The transduced T cells were cultured in 50 units/ml of recombinant human IL-2, IL-7, IL-15 for 10 to 15 days expansion (PeproTech, USA) before functional verification.

### Flow Cytometry

Flow cytometry was performed on a BD FACS Celesta flow cytometer. Data were graphed using FlowJo 7.6. In all cases, cells were washed three times with PBS and then incubated with antibodies for 30 min at 37°C in the dark. Before analysis, cells were washed once and then resuspended in 500 μl PBS. Phenotypic characterization of T cells was determined by directly staining with anti-human CD3-PerCP-cy5.5 (BD Pharmingen, 560835), anti-human CD4-FITC (eBioscience, E10526-1634), anti-human-CD8-APC (eBioscience, 4280528). The CAR-encoded ZsGreen fluorescent protein can be detected by flow cytometry, and the transduction efficiency of T cells was shown by detecting GFP fluorescence on CAR-T cells. The expression of c-Met and PD-L1 on tumor cells was examined by incubating with APC-conjugated anti-c-Met antibody (Sino Biological, 10692-R243-A) and FITC-conjugated anti-PD-L1 antibody (Sino Biological, 10084-R611-F).

### *In Vitro* Cytotoxicity Assay

In the cytotoxicity experiment, Flow cytometry and WB results (**Supplementary Figure 2**) determined five target cells: MHCC97 and HepG2 HCC cell lines with high expression of c-Met and PD-L1, c-Met knockdown HepG2 (HepG2^lo^) cells with low expression of c-Met and high expression of PD-L1, human liver epithelial normal cell LO2 with low expression of c-Met and PD-L1. Next, CP, c-Met, PD-L1, Unrelated CAR-T cells and activated T cells and target cells were co-cultured at the target ratio (E:T) of 10:1, 5:1, and 2:1 at 37° C in a 5% carbon dioxide atmosphere, each group had three repetitions. The co-culture supernatant was collected and analyzed by lactate dehydrogenase analysis (LDH) using a Cytotoxic LDH assay Kit-WST according to the manufacturer’s instructions (DOJINDO, Japan).

### Cytokine Release Assay

The effector cells were co-culture with the target cells at the effect target ratio (E: T) of 10:1 at 37°C in a 5% carbon dioxide atmosphere. The cytokines IL-2 and IFN-γ in co-cultures were detected using ELISA kits according to the manufacturer’s instructions. (Invitrogen, USA).

### Subcutaneous Model of Human Hepatocellular Carcinoma

Six to eight-week-old male nude mice were housed at the Animal Core Facility of Nanjing Medical University under specific pathogen-free conditions. The protocol was approved by the Institutional Animal Care and Use Committee of Nanjing Medical University (Approval NO. IACUC-1804004). For the xenograft model, 1 x 10^7^ HepG2-fLuc cells were suspended in 100μl of culture medium diluted with Matrigel™ (Corning, USA) at a 1:1 ratio and injected into the right flank of each mouse. When the tumor volume reached 200~300 mm^3^, the mice were randomly divided into the following four groups (n = 5): (1) CP CAR-T cell injection; (2) c-Met CAR-T Cell injection; (3) Unrelated CAR-T cell injection; (4) Activated T cell injection. One day before treatment, 200 mg/kg cyclophosphamide was intraperitoneally injected to inhibit the immune system of each mouse. Then, 1 x 10^7^ transduced T cells or active T cells were injected on days 0, 3, and 6 after the tumor model was established.

For the bilateral xenograft model, 1×10^7^ HepG2-fLuc cells were injected into the left flank of each mouse, and a mixture of 5×10^6^ HepG2-fLuc cells and 5×10^6^ HepG2^lo^ cells (fLuc-) were injected into the right flank of each mouse (**Figure 5A**). After 14 days, when the tumor volume reached 200 to 300 mm^3^, the mice were randomly divided into four groups (n = 5). One day before treatment, 200 mg/kg cyclophosphamide was injected intraperitoneally in each mouse to inhibit the immune system. On days 0, 3, and 6 after the tumor model was established, 1 x 10^7^ transduced T cells or active T cells were injected.

### Bioluminescence Assay

To detect tumor sizes dynamically, bioluminescence imaging were performed on fourteen days after HepG2-fLuc cells were injected, 72 h after each treatment and 7 days after the last treatment. For the bilateral xenograft model, Bioluminescence imaging techniques were performed on fourteen days after HepG2 cells were injected and on the 9th day after treatment. Mice were injected intraperitoneally 150 mg/kg D-luciferin (Yeasen Biotech, 40902ES), sedated with isoflurane and imaged using an IVIS Spectrum (PerkinElmer).

### shRNA for c-Met in HepG2 Cells(HepG2lo)

c-Met shRNA (sense primer: 5′-GTCAAGCTTGAATTCCCCA GTGGAAAGACG-3′; antisense primer: 5′-GTCGAATTCAAGCTTCCAAAAAAAATTAGTTCG-3′). The procedure of HepG2^lo^ cells construction was described previously (31). RT-PCR, qRT-PCR and Flow cytometry was used to confirm shRNA lentiviruses that inhibit the expression of c-Met (**Supplementary Figure 3**).

### Western Blot

The protocol of Western blot analysis was described previously (45, 46). The antibodies used were as follows: anti-c-Met (Cell Signaling Technology, 8198S), anti-PD-L1 (Cell Signaling Technology, 13684S), anti-CD3ζ (Santa Cruz Biotechnology, sc-166275), anti-GAPDH (Proteintech, 60004-1- Ig), goat anti-mouse IgG-HRP (FDbio science, FDM007), and goat anti-rabbit IgG-HRP (FDbio science, FDR007). Final detection was performed with an enhanced chemiluminescence system (Tanon, China).

### Immunohistochemistry

Tumor tissue samples were subjected to H&E and immunostained for c-Met (Santa Cruz Biotechnology, sc-514148), Ki-67 (Santa Cruz Biotechnology, sc-23900) as previously described (47, 48). The image was obtained with a microscope (Zeiss, Germany).

### Statistical Analysis

All data are reported as mean ± SD. Statistical analysis was performed using SPSS 20.0. The *t* test and one-way analysis of variance were used, all statistical tests were two-sided, and *p＜*0.05 was considered statistically significant.

## Results

### Construction and Identification of Lentivirus Encoding CP CAR

The construction of CP CAR, c-Met CAR, PD-L1 CAR, and Unrelated CAR was shown in **Figure 1A**. Then the CAR sequences were inserted into a lentiviral expression vector system with green fluorescent protein (GFP). All recombinant plasmid-transfected 293T cells were collected, Western blot was applied to detect the expression of CD3ζ protein which was used as an index of CAR expression. The results indicated that all recombinant plasmid-transfected 293T cells expressed CD3ζ protein (**Figure 1B**). The bispecific CAR contained two scFvs, and the location of CD3 ζ protein in CP CAR transfected cells was close to 80 kD, while the location of monospecific CAR was approximately 50 kD. These results were consistent with the theoretical expectations.

### T Cells Transduction by Lentivirus Encoding CP CAR

T cells transduced by the CP CAR lentivirus were observed by fluorescence microscopy (**Supplementary Figure 1B**), and the number of CAR-T cells or T cells was counted every three days. As shown in **Figure 1C**, compared with transduction levels before, CP CAR-T cells activation was much increased (13.25 ± 0.49) fold, and the amplification of CP CAR-T cells was not significantly different from other groups. The transfection efficiency of T cells expressing CAR was 45.8% to 49.6% (**Figure 1D**). Then, the phenotype of the transduced T cells was determined by flow cytometry, and about 65% of the transduced T cells were CD8^+^ T cells. Only a small proportion of these cells are CD4^+^ T cells (about 25%) (**Figure 1E**). It suggested that T cells were successfully transduced with CP CAR lentivirus.

### CP CAR-T Cells Display Superior Cell Lysis Against HCC Cells *In Vitro*

**Figure 2:**
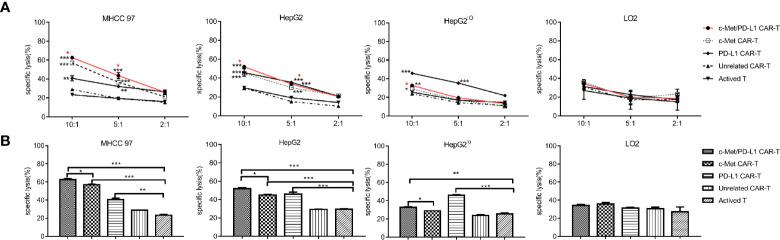
*In vitro* cytotoxicity of CP CAR-T cells against HCC cells. **(A)** Human CAR- T transduced cells or activated T cells were co-incubated with human MHCC97, HepG2 HCC cell lines, human normal liver epithelial cells LO2 and c-Met knockdown HepG2 cells (HepG2^lo^) for 12 h. The cell ratios of effector: target (E: T) was 10:1, 5:1, and 2:1, specific lysis was tested by LDH release assay. **(B)** At the cell ratios of E: T was 10:1, the specific cytotoxicity of different effector cells against various target cells. Data are representative of three independent experiments. Data are shown as mean ± SD. *p < 0.05, **p < 0.01 and ***p < 0.001. (*CP CAR-T cells group compared with activated T cells, *marked with red color means CP CAR-T cells group compared with monovalent c-Met CAR-T cells group).

Before functional verification, the expression of c-Met and PD-L1 on tumor cells was detected by flow cytometry (**Supplementary Figure 2A**) and Western blot (**Supplementary Figure 2B**). As shown in **Figure 2A**, CP CAR-T cells shown higher cytotoxic activities when cultured with c-Met and PD-L1 positive MHCC97, HepG2 HCC cells compared with monovalent CAR-T cells or activated T cells. For HepG2^lo^ cells, the results shown that CP and PD-L1 CAR-T cells shown higher cytotoxic activity, monovalent CAR-T or activated T cells shown little cytotoxic activities. For LO2 cells, CP CAR-T, other monovalent CAR-T or activated T cells exhibited no obvious cytotoxic activities. Then, the fixed E: T ratio was selected, the results shown that the CP CAR-T cells also displayed significant cytotoxic activity for c-Met and PD-L1 positive HCC cells, and its cytotoxic effects were higher than monovalent CAR-T cells (**Figure 2B**). In summary, the results indicated that CP CAR-T cells could specifically kill c-Met and PD-L1 positive HCC cells, but not normal liver epithelial cells. And this cell killing activity is superior to monovalent c-Met CAR-T cells.

### CP CAR-T Cells Produce More Cytokines

**Figure 3:**
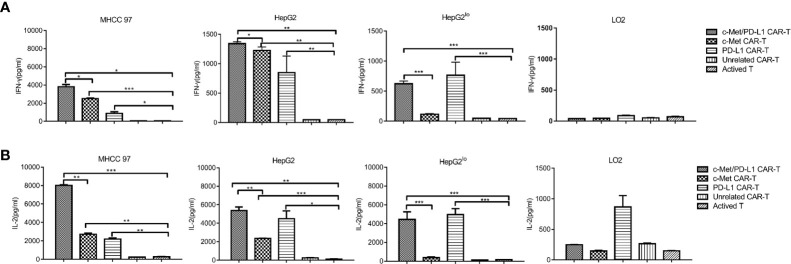
*In vitro* more specific cytokines produced by c-Met/PD-L1 CAR-T cells against HCC cells. At the cell ratios of E: T was 10:1, c-Met/PD-L1, c-Met, PD-L1, Unrelated CAR-T cells or activated T cells were co-incubated with human MHCC97 cells, HepG2 HCC cells, human normal liver epithelial cells LO2 and c-Met knockdown HepG2 cells (HepG2^lo^), for 24 h. **(A)** IFN-γ production was measured by ELISA after the incubation of the CAR- T cells or activated T cells and target cells. **(B)** IL-2 -production was measured by ELISA after the incubation of the CAR- T cells or activated T cells and target cells. Data are representative of three independent experiments. Data are shown as mean ± SD. *p <0.05, ** p <0.01 and *** p <0.001.

Next, cytokine production by CAR T cells in response to target cells were evaluated, it shown the degree of immune response activation. As shown in **Figures 3A, B**, when CP CAR-T cells were co-cultured with c-Met and PD-L1 positive HCC cells, the secretion of cytokines IFN-γ and IL-2 were much higher than that of monovalent CAR-T or activated T cells. When cultured with HepG2^lo^ cells, CP and PD-L1 CAR-T cells shown significant secretion of cytokines IFN-γ and IL-2, and IFN-γ and IL-2 expression was higher than the other groups. When cultured with LO2 cells, all groups shown low levels of IFN-γ and IL-2. The above results displayed that CP CAR-T cells exhibited greater cell-killing ability on c-Met and PD-L1 positive HCC cells than that of the monovalent CAR-T or activated T cells.

### CP CAR-T Cells Significantly Inhibit Tumor Growth of HepG2 Xenografts *In Vivo*

**Figure 4:**
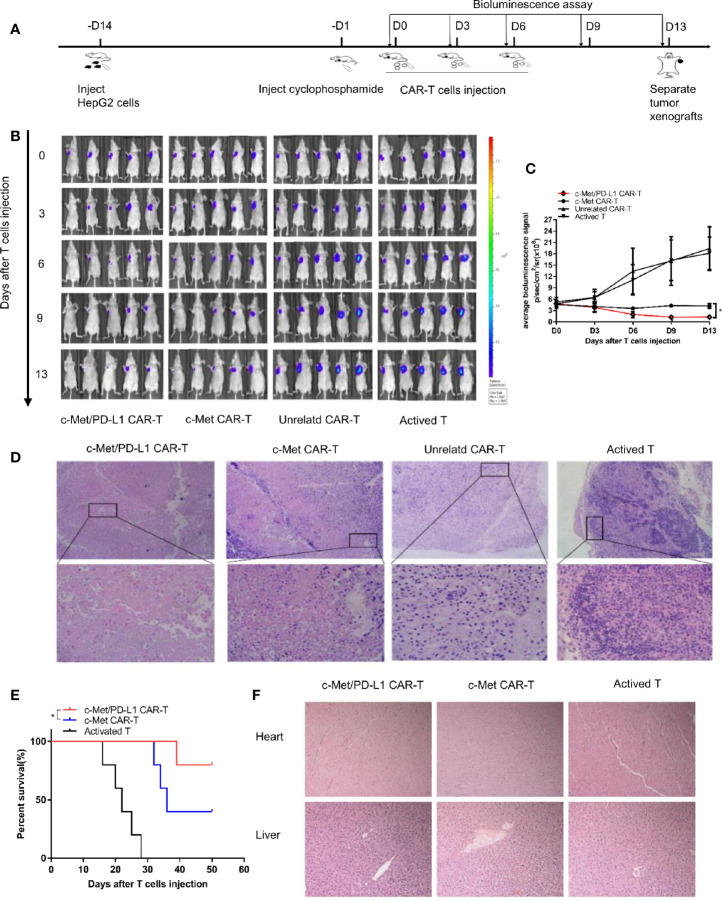
*In vivo* antitumor activities of c-Met/PD-L1 CAR-T cells against c-Met/PD-L1 positive subcutaneous HCC tumors. **(A)** Schematic representation the subcutaneous implantation of HepG2-fLuc cells and treatment of CAR-T cells. **(B)** Mice bearing HepG2-fLuc subcutaneous xenografts progression was assessed by bioluminescence assay on day 0, 3, 6, 9, and 13(n=5). **(C)** Quantification of tumor burden was depicted according to the bioluminescence signal. **(D)** Representative H&E images of tumor xenografts were taken with a microscope under 100x and 200x magnification. **(E)** Kaplan–Meier survival curves of mice treated with CP CAR-T cells, c-Met CAR-T cells and activated T cells (n=5). **(F)** Representative H&E images of normal organ of mice treating with CP CAR-T cells, c-Met CAR-T cells and activated T cells were taken with a microscope under 100x magnification. Data are shown as mean ± SD. *p < 0.05.

To further explore the therapeutic efficacy and safety of bispecific CP CAR-T cells *in vivo*, the xenograft tumor models were constructed, following the process shown in **Figure 4A**. The image results shown observable tumor lysis and even complete tumor elimination in the CP CAR-T cells treatment group, which was superior to the other groups (**Figure 4B**), the tumor burden was much decreased in the CP and c-Met CAR-T cells treatment group (**Figure 4C**). However, the tumor burden in the unrelated CAR-T and activated T cells treatment groups presented much increase. H&E staining demonstrated that the tumor masses in the CP CAR-T cell treated group were highly necrotic, whereas necrotic lesions in the unrelated CAR-T cell-treated group or the activated T cell-treated group were barely detected (**Figure 4D**). As shown in **Figure 4E**, the mice treating with CP CAR-T cells prolonged survival persistence when compared to mice treating with c-Met CAR- T cells. In addition, to further evaluate the safety of CP CAR-T cells, the effects of CP CAR-T cells on the normal organs of mice were determined. HE results revealed the liver and heart of the mice showed no obvious damage after treatment with CP CAR-T cells (**Figure 4F**). Collectively, these results suggest that CP CAR-T cells can specially target and efficiently inhibit the tumor growth of c-Met/PD-L1 positive HCC xenografts. And CP CAR-T cells have a more pronounced therapeutic effects and safety than monovalent CAR-T cells *in vivo*.

### Bispecific CP CAR-T Cells Specially Target Both c-Met and PD-L1

**Figure 5:**
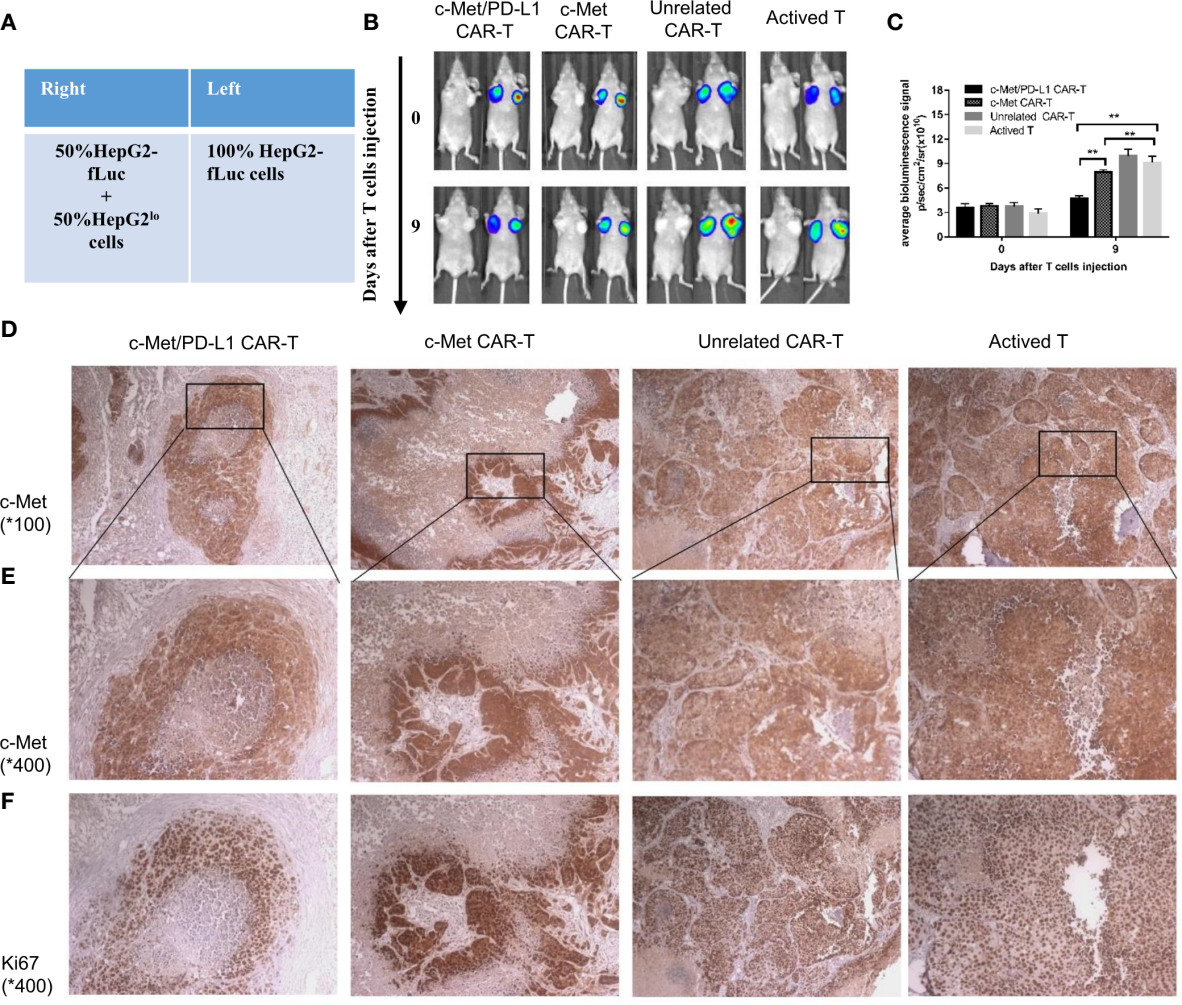
CP CAR-T cells specifically inhibiting c-Met/PD-L1 expression in subcutaneous HCC tumors. **(A)** Schematic showing how to establish a bilateral subcutaneous tumor. **(B)** Tumor progression was assessed by bioluminescence assay on day 0 and 9 after CAR-T cells injection(n=5). **(C)** Quantification of tumor burden was depicted according to the bioluminescence signal. **(D, E)** Immunohistochemical (IHC) staining for anti-human c-Met were performed on tumor samples, representative images were taken with a microscope under 100x and 400x magnification. **(F)** Immunohistochemical (IHC) staining for anti-human Ki-67 were performed on tumor samples, representative images were taken with a microscope under 400x. Data are shown as mean ± SD. **p < 0.01.

To further explore the tumor suppressive effect of bispecific CP CAR-T cells, a bilateral HCC xenograft model was successfully constructed (**Figure 5A**). As shown in **Figure 5B**, the representative left image was under bright field while the right was under bioluminescence signal. Since HepG2^lo^ cells were not constructed to express the luciferase gene, the fluorescence intensity on the left side of the nude mice was higher than that on the right side at day 14 (**Figure 5B**). On the 9th day, the mean fluorescence was found lower in CP and c-Met CAR-T cells treatment group compared to unrelated CAR-T and activated T cells.

However, the fluorescence decrease on the right sides of mice was only observed in CP CAR-T cells treatment group, indicating that CP CAR-T cells have a bispecific killing effect, and its targets are not only for c-Met (**Figures 5B, C**). Then the tumor bulks on the right side of each group mice were isolated, immunohistochemistry was used to detect the expression of c-Met and Ki -67 in the tumor bulks. As shown in **Figures 5D–F**, in all groups, the expression of c-Met and Ki -67 were observed in the same field after serial slices (400x). The outcome shown that CP CAR-T cells treatment group was observed to have the least c-Met and Ki -67 expression levels compared with c-Met, unrelated CAR-T and activated T cells treatment group. The results further suggested that bispecific CP CAR-T cells could specially target c-Met and PD-L1, and that bispecific CAR-T cells had superior anti-tumor effects than monovalent c-Met CAR-T *in vivo*.

## Discussion

Chimeric antigen receptors (CARs) are fusion proteins composed of an extracellular antigen recognition moiety and intracellular T cell activation domains. The ectodomain is usually the single chain variable fragment (scFv) of an antibody specific for a surface molecule on the tumor cell. The intracellular domain ordinarily consists of an activation domain, usually CD3ζ, with costimulatory domains such as CD28, CD137, or CD134 can remarkably promote the survival of CAR-T cells (49, 50). Such a synthetic tumor-targeting receptor can allow transfected T cells to perform antitumor functions in a non-MHC-restricted manner (51). In recent years, the successful application of CAR-T cell immunotherapy in hematological diseases has attracted widespread attention (7, 8). However, the results of CAR-T cells treatment in solid tumors were unsatisfactory. The main concerns for the CAR-T effects in solid tumor are the influence of antigen escape effects (15, 16). Dina et al. constructed a bispecific CAR that target CD19 and CD20 and found that its killing effects on lymphoma cells were better than CD19 monoclonal CAR-T cells (17). In the treatment of glioblastoma, the HER2 and IL-13Rα2 bivalent CAR constructed by Meenakshi et al. showed stronger anti-tumor effects than that of monovalent CAR T cells *in vitro* and *in vivo* (52). Therefore, Bivalent CAR construction is a new strategy to improve “antigen escape effects” to enhance the therapeutic effects of CAR-T in solid tumors.

Studies have shown that c-Met was highly expressed in HCC (18, 19). Nowadays, antibodies against c-Met have also been reported, such as human anti-c-Met high-affinity antibodies (30)and conjugates of Met Fab and doxorubicin (DOX) (31). Our previous studies have shown that human anti-c-Met scFv can target c-Met protein on tumor cells, while c-Met-IgG bound to oxaliplatin can significantly target c-Met positive HCC cells and reduce the side effects of preclinical models of HCC (32). The application for the c-Met Fab patent has been approved in our early work. In addition, c-Met CAR-T has been established in our previous study and have been proved to have a certain killing effect on c-met positive HCC *in vitro* and *in vivo*. However, studies have found that c-Met has mutated in a variety of forms (53, 54), and c-Met inhibitors that target a mutation site tend to be ineffective in subsequent clinical trials (55). Therefore, it is difficult to target the whole HCC tissues by selecting only one antigen as the target of CAR-T therapy. PD-1 is expressed on activated T cells, B cells and myeloid cells, while its ligand PD-L1 is expressed on antigen presenting cells (APC), including monocytes, dendritic cells, and activated B cells (56). Based on previous studies, the PD-L1 expression has been widely considered as a poor prognostic factor for HCC (41, 42). As an immune escape mechanism, the binding of PD-1 and its ligand PD-L1 enhances their role in tumor immune escape. Taken together, c-Met and PD-L1 were selected as the target antigens in this study.

During the study, the bispecific CP CAR was constructed and successfully expressed. Then CP CAR-T cells were obtained follow the protocol and the cell-lysis activities were detected by LDH assay. The results shown that CP CAR-T cells exhibited higher cell-lysis activity for c-Met/PD-L1 positive HCC cells than other monovalent CAR-T or activated T cell groups *in vitro* (**Figure 2A**). In addition, bispecific CP CAR-T cells secreted much higher IFN-γ and IL-2 expression against c-Met and PD-L1 positive tumor cells compared with other groups (**Figures 3A, B**). The results shown that bispecific CP CAR-T cells could target both c-Met and PD-L1 *in vitro*. CP CAR-T cells enhance the cell-lysis activities most likely because it improves the “antigen relapse effects” influence.

*In vivo* experiment, bispecific CP CAR-T cells shown greatest therapeutic effects among all treatment groups (**Figures 4B, C**). H&E staining demonstrated that the tumor masses in the CP CAR-T cell treated group were highly necrotic, which indicated that more tumor cell-lysis was found in CP CAR-T cell treated group *in vivo* (**Figure 4D**). The results shown bispecific CP CAR-T cells had superior anti-tumor effect on the constructed xenograft model. For considering the side effects like cytokine storm and organ damage et al. induced by CAR-T cell therapy, the safety of CP CAR-T cells deserved to be further explored. Firstly, we observed the survival time of mice after CP CAR-T cell treatment was significantly prolonged than that of mice processed with c-Met CAR-T cells, which may benefit from its superior tumor suppressor effects and the potential effects of disturbing PD-1/PD-L1 combination (**Figure 4E**). Besides, HE staining showed that CP CAR-T cells did not cause significant damage to normal organs such as the heart and liver. These experimental results showed that CP CAR-T cells exert superior therapeutic effects and safety (**Figure 4F**). To further illustrate the bispecific CP CAR-T cells could target both c-Met and PD-L1, a bilateral subcutaneous tumor model was constructed (**Figure 5A**). HepG2^lo^ cells were not constructed to express the luciferase gene because this method can tell the difference between HepG2-fLuc and HepG2^lo^ cells. The results shown CP and monovalent c-Met CAR-T cells could specially inhibit the growth of left -side tumors of nude mice. However, only CP CAR-T cells could specially inhibit the growth of the right-side tumor of nude mice. The results indicate that only CP CAR-T cells can specially target PD-L1 *in vivo*. The IHC results shown that the CP CAR-T cells treatment group exhibited a significant decrease in c-Met and Ki-67 levels, which confirmed that the bispecific CP CAR-T cells can improve “antigen relapse effects”.

At present, only little CAR-T therapies in HCC have been reported. The reported targets only including GPC3, NKG2D and CD147 (13, 57, 58). The construction of c-Met CAR has been reported in malignant mesothelioma treatment and has achieved certain anti-tumor effects ***in vivo*** and ***in vitro*** (10). However, the study of c-Met as a CAR-T therapeutic target for HCC has not been reported. In order to solve the antigen relapse problem, this study initially constructed the bispecific c-Met/PD-L1 CAR-T cells, and evaluated the efficacy and safety of HCC treatment *in vitro* and *in vivo*. However, there are still some limitations in this study. Firstly, in order to better simulate the real situation of HCC, an *in situ* model of HCC was significant. In addition, the animals we selected were nude mice, which lacked normal immune function, and other types of mice may be considered for model construction. Since c-Met and PD-L1 antigens selected in this experiment were tumor-associated antigens, which were still slightly expressed on normal hepatocytes, the further safety assessment needs to be further strengthened.

Taken together, these data demonstrate successful construction of CP CAR-T cells. This bispecific CP CAR-T cells specifically target c-Met/PD-L1 positive HCC cells *in vitro* and *in vivo*, and this cytocidal effect is superior to the monovalent CAR-T cells. This study provides an effective strategy for the immunotherapy of HCC in the future.

## Data Availability Statement

The original contributions presented in the study are included in the article/supplementary material. Further inquiries can be directed to the corresponding authors.

## Ethics Statement

The protocol was approved by the Institutional Animal Care and Use Committee of Nanjing Medical University (Approval NO. IACUC-1804004).

## Author Contributions

GJ, QT, and ZF designed and guided this research. WJ, TL, and JG performed the main experiments. JW, LJ, and XS analyzed the data. TY, RJ, and XW performed the supplemental experiments. WJ and TL wrote this manuscript. All authors contributed to the article and approved the submitted version.

## Funding

This study is supported by a grant from the Key Research & Development Program of Jiangsu Province, China (NO. BE2016799) and the National Natural Science Foundation of China (No. 81773268).

## Conflict of Interest

The authors declare that the research was conducted in the absence of any commercial or financial relationships that could be construed as a potential conflict of interest.

## Supplementary Material

Supplementary Figure 1 Detection of CP lentiviral titer. **(A)** Bright field and green fluorescence micrographs (GFP) of 293-T cells transduced with different volumes of CP CAR lentivirus at 100x magnification. **(B)** Bright field and green fluorescence micrographs (GFP) of CP CAR-T cells at 100 x magnification.

Supplementary Figure 2 c-Met and PD-L1 expression on HCC cell lines were detected. **(A)** Expression of c-Met and PD-L1 in various HCC cell lines, human LO2 hepatocytes was examined by flow cytometry. **(B)**Western blot analysis was used to examine the expression of c-Met and PD-L1 in above cells.

Supplementary Figure 3 Validation of the construction of HepG2 cell line transfected with shRNA. **(A)** RT-PCR was used to detect the c-Met expression in HepG2 cell (Blank), HepG2 cell transfected with sh NC or sh c-Met (HepG2^lo^ cell), with GAPDH as loading control. **(B, C)** The expression of c-Met in HepG2^lo^ cell was determined by qRT-PCR and Flow cytometry. Data was presented as Mean ± SD. **p <0.01.
