# Supplementary figures and images for: Bispecific c-Met/PD-L1 CAR-T Cells Have Enhanced Therapeutic Effects on Hepatocellular Carcinoma
Source: Front Oncol. 2021 Mar 10;11:546586. doi: 10.3389/fonc.2021.546586 (PMC7987916; doi:10.3389/fonc.2021.546586)

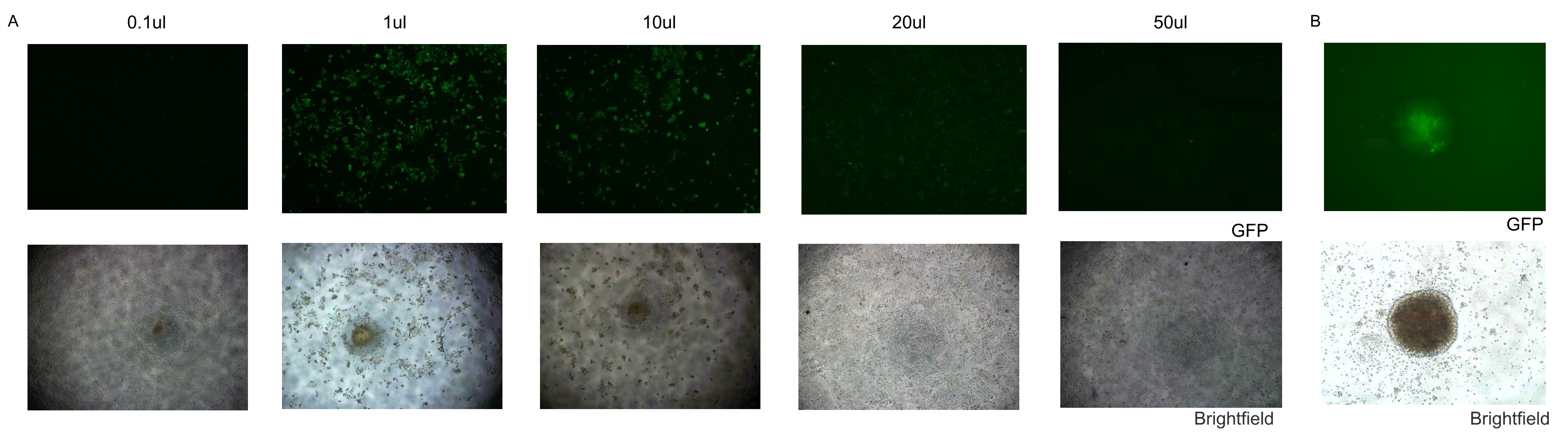

Supplement: Supplementary Figure 1 — Detection of CP lentiviral titer. (A) Bright field and green fluorescence micrographs (GFP) of 293-T cells transduced with different volumes of CP CAR lentivirus at 100x magnification. (B) Bright field and green fluorescence micrographs (GFP) of CP CAR-T cells at 100 x magnification. [file Image_1.tiff]

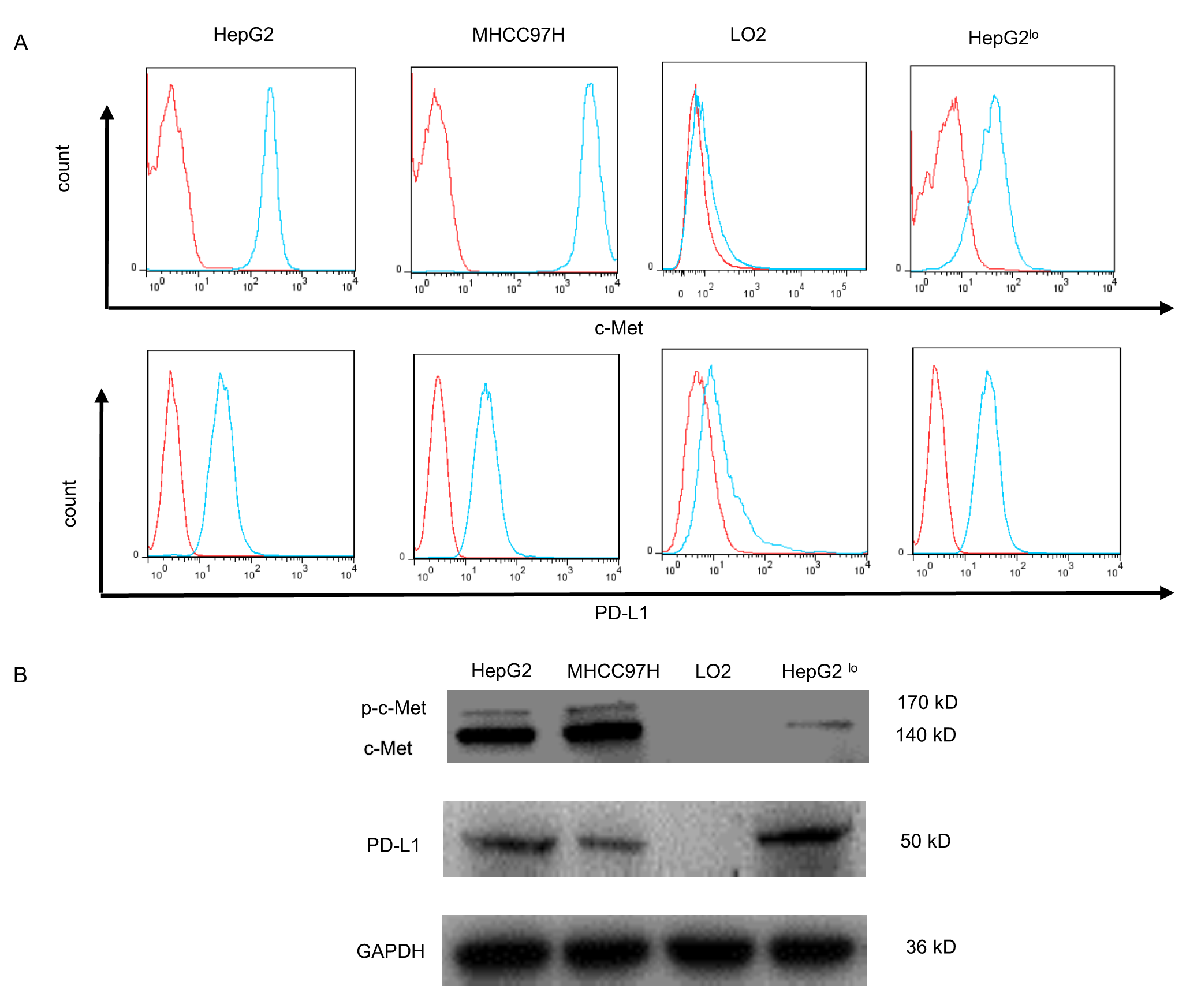

Supplement: Supplementary Figure 2 — c-Met and PD-L1 expression on HCC cell lines were detected. (A) Expression of c-Met and PD-L1 in various HCC cell lines, human LO2 hepatocytes was examined by flow cytometry. (B)Western blot analysis was used to examine the expression of c-Met and PD-L1 in above cells. [file Image_2.tiff]

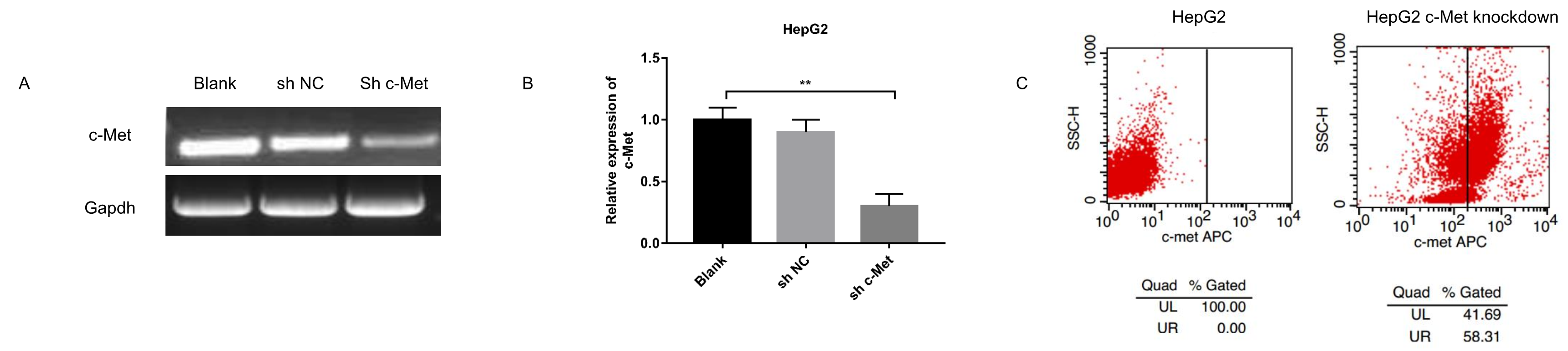

Supplement: Supplementary Figure 3 — Validation of the construction of HepG2 cell line transfected with shRNA. (A) RT-PCR was used to detect the c-Met expression in HepG2 cell (Blank), HepG2 cell transfected with sh NC or sh c-Met (HepG2lo cell), with GAPDH as loading control. (B, C) The expression of c-Met in HepG2lo cell was determined by qRT-PCR and Flow cytometry. Data was presented as Mean ± SD. **p <0.01. [file Image_3.tiff]
