# Supplementary material for: Bispecific c-Met/PD-L1 CAR-T Cells Have Enhanced Therapeutic Effects on Hepatocellular Carcinoma
Source: Front Oncol. 2021 Mar 10;11:546586. doi: 10.3389/fonc.2021.546586 (PMC7987916; doi:10.3389/fonc.2021.546586)
Supplement: Supplementary file 4 [file Table_1.docx]

Supplementary Table 1**.** Amino acid sequences for c-Met, PD-L1 scFv light and heavy chains.

A

| Amino acid sequence for c-Met scFv light chain | GVGLVGSGGGVVGPGASLALSCAASGPTPSSTAMHTVAGAPGLGLGTVAVITTAGSALTTAASVLGAPTISAAASLATLTLGMASLAAGATAVTTCAAAATGPATTGGGTLVTVSP |
| --- | --- |
| Amino acid sequence for c-Met scFv heavy chain | GLGMTGSPSLLSASTGAAVTISCAASGSISSTLATTGGLPGLAPLLLITAASSLGSGVPSAPSGSGSGTAPTLTISSLGPGAPATTTCGGSTSTPHTPGGGTLLGILA |

B

| Amino acid sequence for PD-L1 scFv light chain | GVGLLGSGGGLVGPGGSLALSCAASGPTPSSTIMMTVAGAPGLGLGTVSSITPSGGITPTAASVLGAPTISAAASLATLTLGMASLAAGATAVTTCALALLGTVTTVATTGGGTLVTVSS |
| --- | --- |
| Amino acid sequence for PD-L1 scFv heavy chain | GSALTGPASVSGSPGGSITISCTGTSSAVGGTATVSTTGGHPGLAPLLMITGVSAAPSGVSAAPSGSLSGATASLTISGLGAGAGAATTCSSTTSSSTAVPGTGTLVTVL |
